# Supplementary material for: Genetic comparisons of interleukin-17 reveal a framework for complex signaling evolution
Source: bioRxiv. 2026 Apr 14:2026.04.13.718218. Preprint. [Version 1] doi: 10.64898/2026.04.13.718218 (PMC13105070; doi:10.64898/2026.04.13.718218)
Supplement: Supplement 3 [file media-3.zip › Supplemental files for paper/ERC/IL17xIL17_comparisons/IL25_IL17RB.pdf]

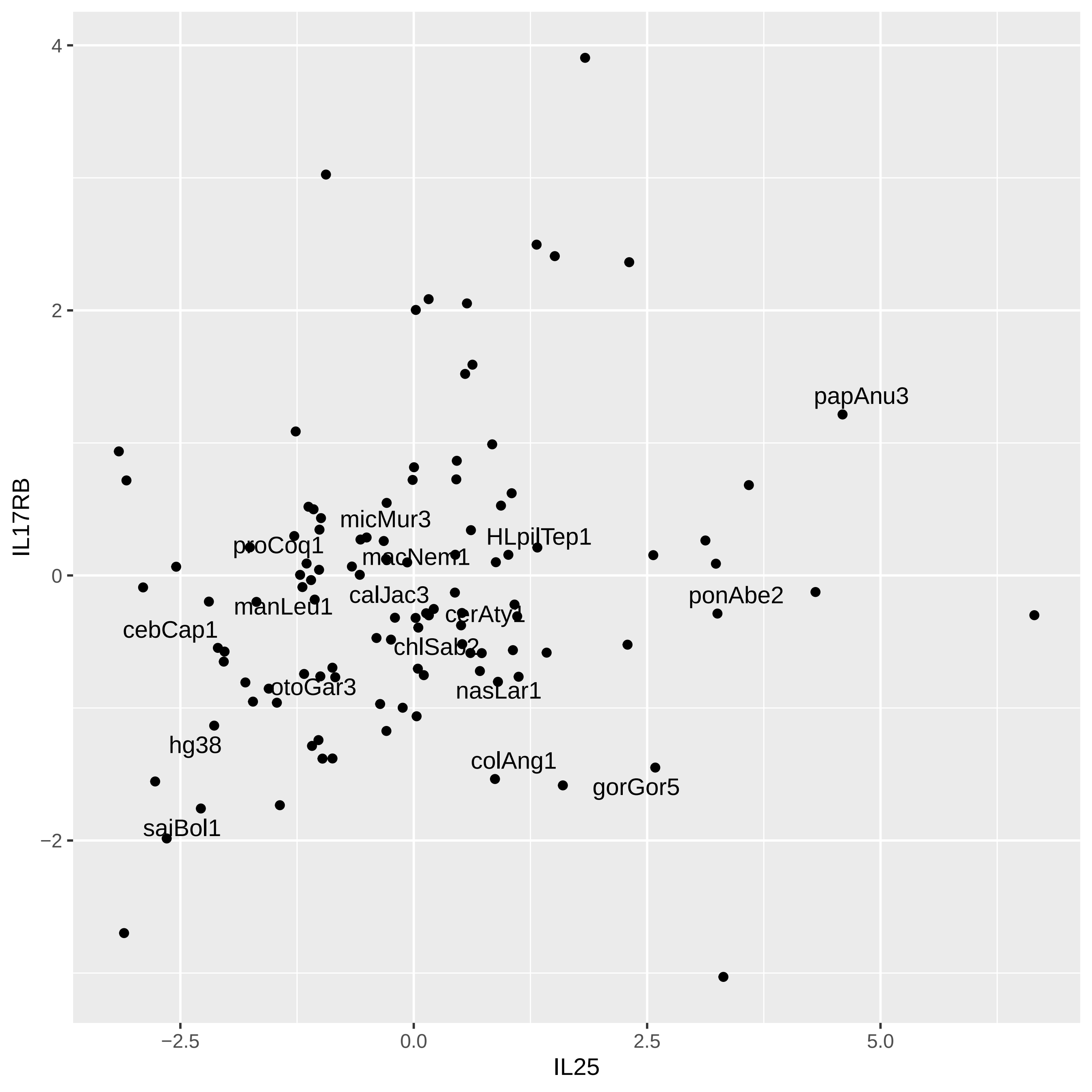

papAnu3
micMur3
HLpilTep1
proCoq1
macNem1
calJac3
ponAbe2
manLeu1
cerAty1
cebCap1
chlSab2
otoGar3
nasLar1
hg38
colAng1
gorGor5
saiBol1
4
2
0
−2
−2.5
0.0
2.5
5.0
IL17RB
IL25
